# Bulk Tea Shoot Detection and Profiling Method for Tea Plucking Machines Using an RGB-D Camera

**DOI:** 10.3390/s25237204

**Published:** 2025-11-25

**Authors:** Yuyang Cai, Xurui Li, Wenyu Yi, Guangshuai Liu

**Affiliations:** 1 School of Mechanical Engineering, Southwest Jiaotong University, Chengdu 610031, China; cyy2023@my.swjtu.edu.cn (Y.C.); motorliu7810@swjtu.edu.cn (G.L.); 2 School of Mechanical Engineering, Sichuan University, Chengdu 610065, China; xuruili@scu.edu.cn; 3 Yibin Institute of Industrial Technology, Sichuan University Yibin Park, Yibin 643000, China; 4 Key Laboratory of Agricultural Equipment Technology for Hilly and Mountainous Areas, Ministry of Agriculture and Rural Affairs, Chengdu 610066, China

**Keywords:** bulk tea plucking, agricultural machinery, profiling, RGB-D camera, super-green feature

## Abstract

Due to the shortage of rural labor and an increasingly aging population, promoting the mechanized plucking of bulk tea and improving plucking efficiency have become urgent problems for tea plantations. Previous bulk tea plucking machines have not fully adapted to tea plantations in hilly areas, necessitating enhancements in the performance of cutter profiling. In this paper, we present an automatic cutter profiling method based on an RGB-D camera, which utilizes the depth information of bulk tea shoots to tackle the issues mentioned above. Specifically, we use improved super-green features and the Otsu method to detect and segment the shoots from the RGB images of the tea canopy taken from different lighting conditions. Furthermore, the cutting pose based on the depth value of the tea shoots can be generated as a basis for cutter profiling. Lastly, the profiling task is completed by the upper computer controlling motors to adjust the cutter pose. Field tests were conducted in the tea plantation to verify the proposed profiling method’s effectiveness. The average bud and leaf integrity rate, leakage rate, loss rate, tea making rate, and qualified rate were 81.2%, 0.91%, 0.66%, and 90.4%, respectively. The results show that the developed algorithm can improve cutting pose calculation accuracy and that the harvested bulk tea shoots meet the requirements of machine plucking quality standards and the subsequent processing process.

## 1. Introduction

Tea is a prominent non-alcoholic beverage in today’s society [1,2]. More than 3 billion people in 160 countries and regions like to drink tea. The global tea market was valued at nearly 200 billion U.S. dollars in 2020 and is expected to rise to over 318 billion U.S. dollars by 2025 [3]. According to the processing techniques, there are six categories of tea: green, yellow, white, oolong, black, and dark tea [4]. Tea can be categorized into bulk tea and famous tea. Bulk tea refers to products mainly sold in bulk, which are relatively inexpensive and produced in large quantities [5]. At present, bulk tea is mainly plucked by mechanized production methods with the standard of “a bud with two leaves” and “a bud with three leaves” [6]. Tea plucking machines for bulk tea adopt the “one size fits all” standard, which uses a reciprocating cutter to cut the entire tea canopy at a certain depth [7]. When plucking bulk tea in tea plantations located on hilly terrains, the cutter requires real-time pose adjustments to adapt to the sloping terrain and the uneven growth of tea canopies. Therefore, the key to bulk tea harvesting is the cutter profiling of the tea canopy.

**Figure 1:**
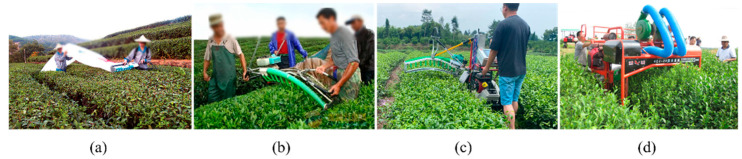
Structures of tea plucking machine: (**a**) single hand-held, (**b**) double-carried, (**c**) self-propelled and (**d**) track riding type.

In recent years, there has been an emergence of tea plucking machines for bulk tea. Figure 1 shows the structures of the tea plucking machine with single-carried, double-carried, self-propelled, and track riding type [8]. The single-carried tea plucking machine has simple structure and convenience, but it is not suitable for large-scale tea plantations due to low operating efficiency [9]. The double-carried tea plucking machine is the most prevalent, accounting for about 60% of the tea plucking machines used in China. However, the double-carried tea plucking machine requires two people to operate the machine and two people to coordinate the tea collecting bag, which is a high labor cost. Meanwhile, the noise, vibration, and hot gas emission during the oil-powered trimmer’s operation easily fatigues the operator. The combination of the self-propelled mobile platform and tea tree trimmer through a cantilever structure is the basic composition of the self-propelled tea plucking machine, which has the advantages of strong practicability and low cost [10]. When the tea plucking machine with a riding type travels along the tracks on both sides of the tea rows, the cutter moves on the top of tea trees, which is suitable for tea plantations in the plains as well as on gentle slopes in the hills (below 15°) [8]. Domestic tea plantations are primarily distributed in hilly areas characterized by intricate topography. Most tea plantations lack tractor roads and have specific slopes, making it challenging for tea plucking machines of riding type to adapt to such landscapes. Ensuring the cutter’s flexible adjustment is imperative because the cutter needs to be adjusted before each harvest to fit the tea canopy. A self-propelled tea plucking machine can adjust the height of the cutter by manually rotating the mechanical lifting mechanism to adapt to different heights of the tea canopy [11]. The electric lift adjustment simplifies the operator’s manual adjustment of the cutter height by controlling it with two operating buttons [12]. Nevertheless, the operation of driving the machine and manual adjustment of the lifting mechanism have certain drawbacks, including slow response times and inconvenient operation.

By achieving adaptive profiling of the tea canopy, the automation level of mechanized bulk tea plucking can be significantly enhanced. The adaptive profiling of the tea canopy means that the arc-shaped cutter can change the cutting pose by recording the change data of the contour of the tea tree top. The profiling process can be implemented through mechanical contact structure, non-contact sensor detection, and machine vision. Two steel sheets connected with the angle sensor on the tea canopy could obtain the angular displacement, combined with the PLC control motor to adjust the rising and falling of the cutter automatically [13]. On the contrary, the change in contact force easily affected the mechanical contact structure. Tang et al. separated the tea shoots and the cutter through computer vision algorithms to detect the tea shoots’ pixel size to control the cutter’s height [14]. However, the cutter adjustment position of this design solution was limited [15]. Zhao et al. developed a distributed controlled riding tea plucking machine, which used an ultrasonic sensor to sense the distance between the tea canopy and the cutter [16]. Screw rod transmission was used to realize the profiling of the tea canopy. Significantly, the highly precise non-contact sensor could not distinguish the tea shoots from the tea canopy, so the old leaves would influence the obtained data. Using an RGB-D camera to segment tea shoots and considering the depth of the tea shoots as the basis for height and angle adjustment of the cutter can avoid the disadvantages of the above methods.

Tea shoot detection is an essential part of the tea plucking process. Color and shape features are often used to detect tea shoots traditionally. Tang et al. analyzed the thresholds of G and G-B components to distinguish tea shoots from the image by the improved Otsu algorithm [14]. Wang et al. used an improved algorithm based on color and regional growth to divide the tea shoots in the images [17]. According to the model database, the characteristic parameters of tea shoots were extracted to complete the three-dimensional modeling of tea shoots. Zhang et al. proposed a new tea shoot recognition and segmentation method based on an improved watershed algorithm, which used B and G-B components to improve the differentiation degree of old leaves and tea shoots [7]. Qin et al. used the R-B components to identify the buds of the famous tea and used Otsu for threshold segmentation. The processing time was 0.288 s, and the average misidentification rate was 28.7% [18]. Significantly, the differentiation between a single bud without leaves and a bud with one leaf could not be accomplished by color features alone. There also would be a certain degree of false recognition due to light effects. It can be seen that traditional detection methods based on color and shape features can be applied to recognize tea shoots with short processing time and certain recognition accuracy. Deep learning techniques have recently emerged as powerful methods for learning feature representations automatically from data, which have improved objection detection significantly [19]. Ji et al. proposed an apple recognition method based on improved YOLOv4, which can locate and recognize apples in various complex environments [20]. Xu et al. presented a detection and classification approach based on the YOLOv3 and DenseNet201 network, and the results showed that the recognition accuracy of tea shoots reached 95.7% for the images shot from the side [21]. Gui et al. applied the Yolo-Tea object detection algorithm to tea bud detection and improved tea bud recognition in unstructured environments [22]. Xie et al. proposed Tea-YOLOv8s for tea bud detection, integrating data augmentation, DCNv2, GAM attention and SPPFCSPC. It achieved 88.27% mAP@0.5, outperforming YOLOv8s by 3.59 percentage points in tea bud detection [23]. Fang et al. developed lightweight TBF-YOLOv8n for tea bud detection, with DSCf, CA attention, SIOU loss and DySample. It reached 87.5% precision and 85.0% mAP50 for tea buds, reducing GFLOPs by 44.4% vs. YOLOv8n [24]. It can be perceived that the deep learning method has better recognition accuracy and is the critical technology for famous tea plucking.

**Figure 2:**
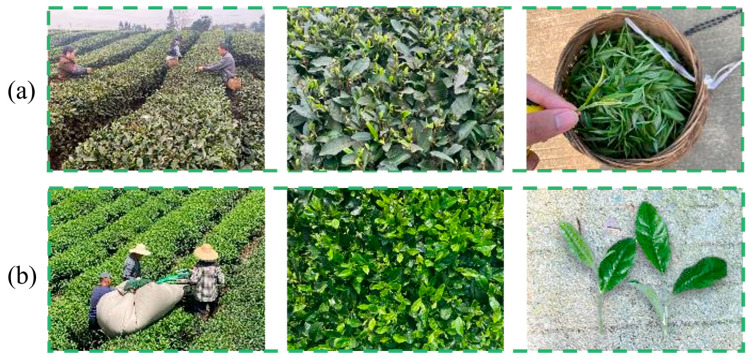
Different types of tea: (**a**) famous tea in the tea canopy and plucked famous tea, and (**b**) bulk tea in the tea canopy and plucked bulk tea.

In summary, in the field of tea shoot detection, both traditional image processing methods and deep learning methods have been widely applied. However, famous tea and bulk tea exhibit distinct characteristics, as shown in Figure 2. The famous tea shoots are scattered within the tea canopy and exhibit significant differences in both color and shape features compared to the older leaves. In contrast, the bulk tea shoots are distributed across the entire surface of the tea canopy, exhibiting significant color differences compared to the old leaves, while their shape characteristics are similar. If deep learning is used to detect bulk tea only based on color differences, there will be false detections and missed detections. In this study, image processing aims to acquire depth information of tea shoots and provide a basis for cutter profiling. Tea shoot detection can minimize the impact of depth values from old leaves and backgrounds, enhancing the accuracy of cutting pose calculation. Therefore, a bulk tea shoot detection algorithm under different weather conditions based on color features and threshold segmentation is developed to segment bulk tea shoots.

The study includes the following parts: (i) detection and segmentation of bulk tea shoots in RGB images captured by RGB-D cameras under different lighting conditions using improved super-green features and the Otsu method, and extraction of the valid depth information corresponding to the young shoot regions in the tea canopy based on the segmented images. (ii) A cutter profiling method is designed to define and calculate the cutting pose based on the depth information of the bulk tea shoots. (iii) A field test is conducted to verify the effectiveness of the designed tea plucking system.

The remainder of this paper is organized as follows. Section 2 presents the bulk tea shoot detection algorithm and details the cutter profiling method and control scheme. Experiment results and discussion are provided in Section 3. Finally, conclusions are drawn in Section 4.

## 2. Materials and Methods

### 2.1. Overview of the System

**Figure 3:**
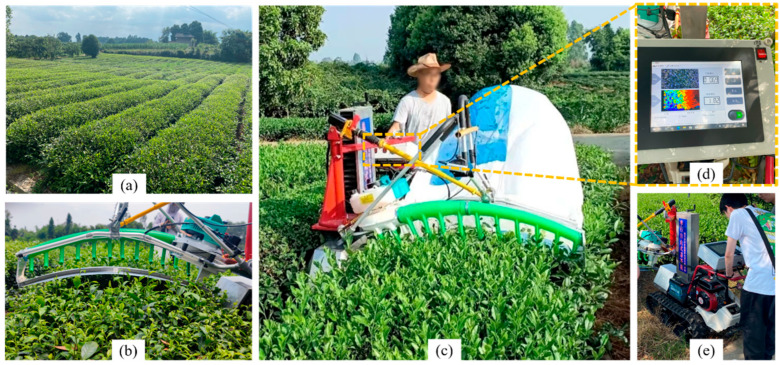
Bulk tea plucking system: (**a**) experimental tea plantation used in this study, (**b**) the arc-shaped cutter and tea canopy, (**c**) the tea plucking machine with an operator, (**d**) human-machine interface, and (**e**) the crawler chassis.

In a tea plantation, tea rows are closely arranged with each other. The upper surface of the tea canopy was arc-shaped and similar in shape to the cutter (Figure 3b). The sloping terrain in hilly regions, coupled with uneven growth of tea canopies resulting from non-standard management of tea plantations, often poses certain challenges to mechanized tea plucking. To adapt to the environment of tea plantations in hilly areas, we presented the development of a self-propelled tea plucking prototype. The crawler chassis was employed as the walking and support platform for the tea trimmer. Compared to wheeled chassis, the tracked chassis offered advantages such as lower ground pressure and smoother movement (Figure 3e). The control unit and human-machine interface were positioned above the throttle adjustment, facilitating the operator’s observation. An Intel RealSense D435i RGB-D camera, manufactured by Intel Corporation (Santa Clara, CA, USA), was located above the center of the cutter and moved with it. The mounting position was in front of the cutter to capture the bulk tea shoots vertically.

**Figure 4:**
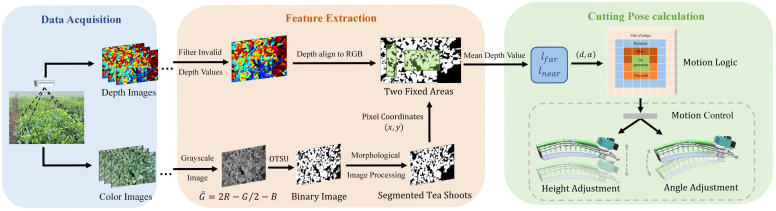
Overview of bulk tea shoot detection and cutter profiling process.

We have designed a cutter profiling method for the crawler self-propelled tea plucking machine to achieve the cutter’s adaptive profiling of tea canopies. The workflow is shown in Figure 4. First, the RGB-D camera acquired real-time RGB and depth images of the tea tree’s surface. Secondly, the average depth values of bulk tea shoots were obtained using an image processing algorithm based on super-green features and Otsu. Additionally, depth images were smoothed and abnormal depth values from tea leaves with holes and dense weeds were filtered. Finally, the motor moved and rotated the cutter based on the cutting pose, cutting the bulk tea shoots to the desired depth.

### 2.2. Visual Perception

The depth information of bulk tea shoots in the tea tree top is an effective basis for controlling the cutter. The images captured by the depth camera include bulk tea shoots, old leaves, and the background. The newly grown bulk tea shoots are typically located at the top of the tea trees and distributed across the entire upper surface of the tea canopy. When the depth camera captured images vertically from above the tea tree top, the bulk tea shoots were densely stacked on each other. The older leaves beneath the bulk tea shoots appear darker in the RGB space due to the shading effect, facilitating the detection of the tea shoots in RGB space. To elaborate, an RGB image consists of three independent color channels: Red (R), Green (G), and Blue (B). Each channel represents the intensity of its respective color, with pixel values ranging from 0 to 255. Fresh bulk tea shoots, with their vivid green hue, have significantly higher G channel values than R and B channels. In contrast, shaded older leaves have lower values in all three channels, particularly the G channel, which is why they appear darker. An approximate depth of the tea canopy’s upper layer could be obtained by filtering out depth outliers. However, the depth values of the older leaves significantly contribute to the deviation of the mean depth from the surface of the tea canopy. Therefore, a bulk tea shoot detection algorithm based on the super-green features and Otsu was used to segment tea shoots from old leaves and backgrounds to obtain the average tea shoot depth robustly.

**Figure 5:**
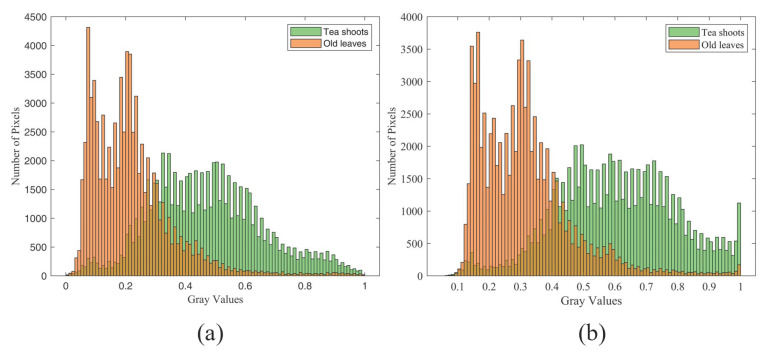
Component of the gray histogram about tea shoots and old leaves: (**a**) R component, and (**b**) G component.

**Figure 6:**
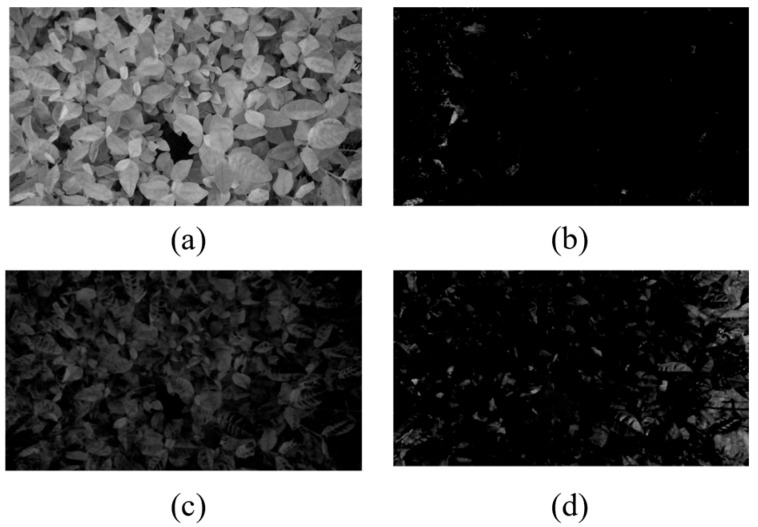
Grayscale conversion in the different combinations of R, G, and B channels: (**a**) 2G−R/2−B, (**b**) 2R−G−B, (**c**) G−B and (**d**) 2B−G−R.

The color images captured by the RGB-D camera are converted into grayscale images, and the gradients of its pixel points will be used for bulk tea shoot detection. The salience regions of the image need to be adjusted before the conversion process to minimize interference from the image area outside the tea shoots. By separating the RGB model image channels, it was found that there were significant differences in G and R components between bulk tea shoots and old leaves. This aligns with the inherent characteristics of the RGB channels: fresh tea shoots retain high G channel intensity due to their green pigment, while old leaves have reduced G channel values and relatively balanced R/B channel distribution, leading to the observed differences. Figure 5a and Figure 5b correspond to the gray histogram of the R component and G component of tea shoots and old leaves, respectively. Moreover, the color green has the following characteristics in the model: for components R, G, and B of the same pixel, there is always G greater than R and B. After separating the R, G, and B channels of the color image, the gray value of the image can be changed by superimposing and subtracting the grayscale values of the R, G, and B channels [25]. Combined with the above features, the role of R and G components in color space was highlighted by increasing the value of G and R points. The $2G-\left(R/2\right)-B$ index was superior to other indices (2R−G−B, G−B and 2B−G−R) (Figure 6).

(1) $$\bar{G}=2G-\left(R/2\right)-B$$

**Figure 7:**
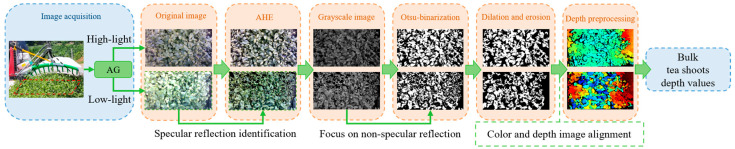
The bulk tea shoot detection algorithm used in different lighting conditions.

The collected images in the field were influenced by factors such as changing outdoor natural lighting conditions and uncertainties in weather, making the gray-level differences between bulk tea shoots and older leaves less stable under super-green features. As shown in Figure 7, the images under different lighting conditions were divided into two grayscale ranges based on average grayscale (AG) values: [90, 140) and [140, 220], representing low-light and high-light images, respectively. The AG value refers to the average grayscale of the tea canopy region. The thresholds 90 and 140 were determined by statistical analysis of typical field images under different weathers. The AG values of the low-light images obtained in overcast and cloudy weather were similar and could be classified together for bulk tea shoot detection. However, under the clear sky and direct sunlight conditions, the high-light images required additional processing to handle the specular reflection in the images.

**Figure 8:**
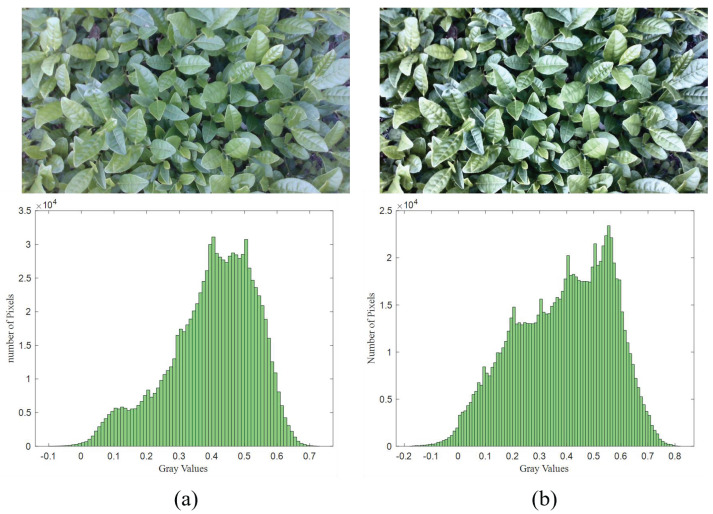
Adaptive histogram equalization image enhancement processing: (**a**) the original image and the grayscale histogram, and (**b**) the image and histogram after image enhancement processing.

For low-light images, contrast limited adaptive histogram equalization was applied to enhance the color contrast between bulk tea shoots and old leaves. CLAHE was configured with a clip limit of 2.0 and a tile grid size of 8 × 8 pixels. As shown in Figure 8, the grayscale histogram of the enhanced image showed a bimodal distribution, which was beneficial for Otsu thresholding. Then, the image was converted to grayscale using Equation (1) and binarized with the threshold value taken by Otsu [26]. The calculation process of OTSU thresholding is as follows: iterate through each gray level in the image as the threshold, divide the gray levels into two categories according to the threshold, calculate the between-class variance after each classification sequentially, and select the gray level corresponding to the maximum between-class variance as the optimal threshold. After the threshold was determined, the gray image was binarized, and the bulk tea shoot segmentation was almost completed. However, there are still some remaining noise or residual old leaves affected by factors such as illumination. Therefore, it is still necessary to perform mathematical morphological processing on the binary image to remove noise and retain the main contours of young leaves. Morphological erosion was employed to remove isolated small regions and obtain the pixel coordinates of the retained regions. As for high-light images, the specular reflection areas were first identified, and their grayscale values were set to 0 to avoid interference with the grayscale conversion of the super-green index. Additionally, during the Otsu calculation, the pixels with a gray value of 0 were excluded to focus solely on non-specular reflection areas. After completing the segmentation of bulk tea shoots, the depth image was aligned with the RGB image according to the camera’s intrinsic parameters, and the corresponding depth information could be obtained directly through the pixel coordinates in the RGB image. Due to external factors such as mechanical vibrations and lighting conditions, it was essential to perform preprocessing on the depth map. For example, depth values for holes or outliers can be filtered by setting thresholds. The median filtering process could suppress noise and smooth the image. Finally, the preprocessed depth values of the bulk tea shoots served as the basis for adjusting the cutting pose. Significantly, since the tea shoot detection aims to acquire depth information rather than precise positioning, it is unnecessary to fully segment the tea shoots. Therefore, the focus of the study is not on issues such as the overlapping and occlusion of tea leaves.

### 2.3. Cutter Profiling Method

**Figure 9:**
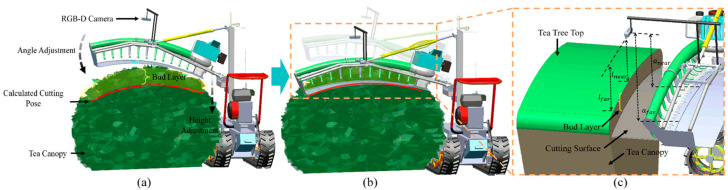
The profiling process: (**a**) the cutter deviates from the bud layer and needs to be moved to the calculated cutting pose by adjusting the angle and height of the cutter, (**b**) the cutter is aligned with the surface of the tea tree top and located at a certain distance below, and (**c**) the spatial relationship between the cutter and the tea canopy.

The cutter needs to be flexibly adjusted with the tea canopy for a tea plucking machine. The automatic cutter profiling method of tea plucking through machine vision is similar to grasping objects by a manipulator, which requires calculating the cutting pose and moving the cutter to the bottom of the bud layer in the tea canopy (Figure 9). The region extending to a certain depth below the surface of the tea canopy constitutes the bud layer, with this depth corresponding to the plucking depth required by the tea plantation.

**Figure 10:**
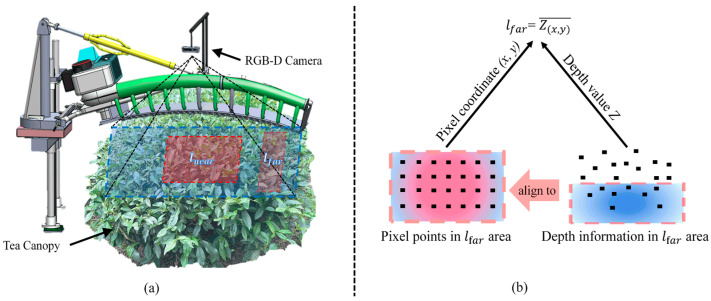
Calculate the average depth values of all pixel points within two fixed regions for $l_{near}$ and $l_{far}$: (**a**) two fixed regions in the RGB image, and (**b**) the calculation process of $l_{far}$: after obtaining the coordinates of all pixels in the $l_{far}$ region and their corresponding depth values, the average value is taken as $l_{far}$.

When the machine was running between tea rows, the top surface of the tea tree was directly below the depth camera. As shown in Figure 9c, the cross-section of the upper surface of the tea canopy in the two-dimensional vertical plane approximates a symmetrical arc, which is similar to the shape of a reciprocating cutter. The cutting pose can be calculated by the vertical distance ($l_{near}$ and $l_{far}$) between the upper surface of the tea tree and the depth camera. Since using a single pixel to calculate the vertical distance may bring errors, the average depth value of the pixel points of all the bulk tea shoots within a fixed position and range is taken to calculate the value of $l_{near}$ and $l_{far}$ (Figure 10). $l_{near}$ and $l_{far}$ represent the mean depths of tea shoots within the central and left regions of the tea canopy image, respectively. In this study, the depth camera was installed on the cutting tool for obtaining RGB-D data and moved with it, which meant the camera’s position concerning the cutter ($a_{near}$ and $a_{far}$) was fixed and known. $a_{near}$ and $a_{far}$ respectively represent the vertical distances from the central and left sides of the cutter to the installation position of the depth camera. The ideal cutting pose is that the cutter matches the upper surface of the tea canopy and is located at a certain distance below the canopy, resulting in the ideal length of harvested bulk tea shoots. The 2D pose of the cutter relative to the tea tree top can configure the cutting pose $\left(d,\alpha \right)$. d is the height adjustment value required for the tea shoots cut in the middle region to satisfy the desired length such that the value of $a_{near}-l_{near}$ is within the allowable range. α is the angle adjustment value required for the tea shoots cut in the left region to satisfy the ideal length such that the value of $a_{far}-l_{far}$ is within the permissible range.

**Figure 11:**
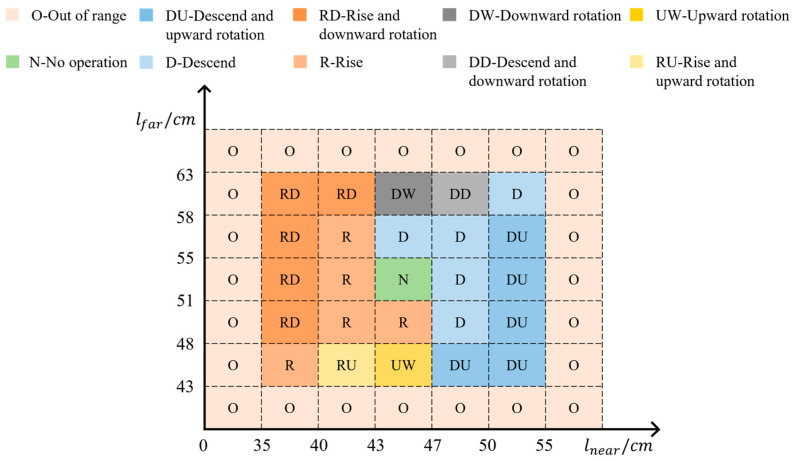
The logic of cutter motion based on $l_{near}$ and $l_{far}$.

The ultimate goal of the cutting pose calculation is to find the relationship between $l_{near,far}$ and $a_{near,far}$, which determines d and α. The difference between $l_{near}$ and $a_{near}$ exceeding the ideal length of the bud layer means that the current height of the cutter deviates from the ideal cutting pose, and the height of the cutter needs to be changed. When $l_{far}$ and $a_{far}$ differ widely, the cutter is not aligned with the tea tree top, and the cutter needs to be rotated so that all bulk tea shoots cut by the cutter conform as well as possible. After fixing the installation position of the camera, the vertical distance $a_{near}$ and $a_{far}$ from the depth camera to the center and left side of the arc-shaped cutter can be measured. Compared with $l_{near}$ and $l_{far}$, the resulting motion logic is shown in Figure 11. The motion of the cutter can be divided into ten types: out of range, rise, no operation, upward rotation, etc. The range of $l_{near}$ and $l_{far}$ in each motion type is customized according to the actual factors, such as the height and width of the tea canopy and cutter parameters. The value ranges of $l_{near,far}$ are assigned to each motion type, considering harvesting objects, cutter operation range, and camera installation position. Furthermore, the $l_{near}$ and $l_{far}$ calculated in real time during the tea plucking process correspond specifically to a particular motion type.

**Figure 12:**
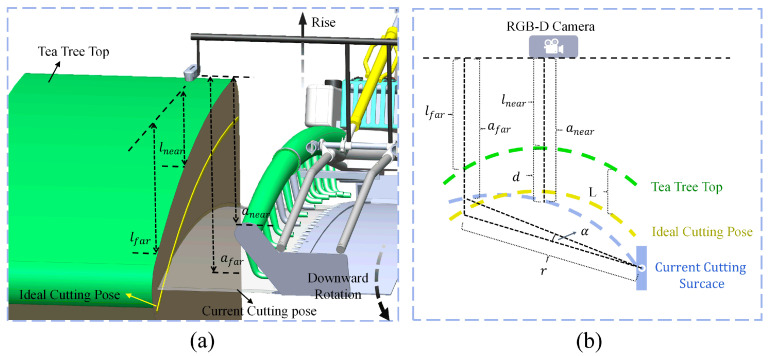
Schematic diagrams: (**a**) the motion logic of rise and rotate downward, and (**b**) calculation principle of α  and d.

In this study, the tea plantation requires the plucking length of bulk tea young shoots to be approximately 10 cm. Therefore, the cutter needs to penetrate about 10 cm into the tea canopy to cut the young shoot layer. Meanwhile, the depth camera fixed on the cutter has a fixed position relative to the cutter, with measured values of $l_{near}=55$ cm and $l_{far}=61$ cm. The depth camera captures RGB-D data of the tea canopy, and real-time values of $l_{near}$ and $l_{far}$ are calculated by the host computer. When the cutter penetrates into the young shoot layer in the ideal cutting pose, the distances between the upper surface of the tea canopy and the depth camera namely $l_{near}$ and $l_{far}$ are approximately 45 cm and 51 cm respectively. Therefore, when $l_{near}=43$ to 47 cm and $l_{far}=51$ to 55 cm, the cutter is located 10 cm below the upper surface of the tea canopy without angular displacement, so no adjustment of the cutter pose is required, that is, no operation. Following the above logic, the threshold ranges of $l_{near}$ and $l_{far}$ required for other pose adjustment logics can be formulated. Taking the case in Figure 12a as an example, when $l_{near}=35$ to 40 cm and $l_{far}=51$ to 55 cm, due to the influence of sloping terrain or changes in the upper surface of the tea tree, the cutter is lower than the tea canopy and has a certain amount of offset. The cutter cuts the tea shoots too long in the middle region and too short in the left region, which means it needs to be moved up and rotated downward to achieve the desired pose.

When the cutter pose needs to be changed, d and α should be calculated to send instructions to the motor. Taking Figure 12a as an example, two actions will be carried out simultaneously. As shown in Figure 12b, d is the vertical distance between the tea canopy and the cutter in the current state. It can be obtained that

(2) $$d=\left(a_{near}-l_{near}\right)-L,$$

where L is the expected length of the bud layer. Two relays are connected between the DC motor and the motion controller to control the rise and fall of the cutter. The upper computer establishes communication with the controller by the Ethernet, which controls the energizing time of the relays to realize the height adjustment of the cutter. α is the angle at which the cutter needs to be rotated so that the bulk tea shoots cut in the left region meet the requirements. α is approximately calculated as follows:

(3) $$\alpha =\left(\left|L-\left|a_{far}-l_{far}\right|\right|\times 180\right)/r\cdot \pi ,$$

where r is the fixed distance from the rotation point of the cutter’s bracket to the left side of the cutter.

**Figure 13:**
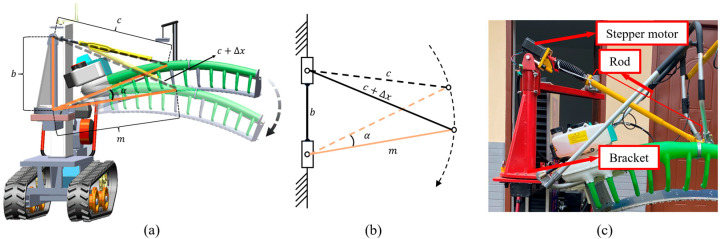
Calculation principle of Δx: (**a**) 3D model, (**b**) skeleton of mechanism and (**c**) physical map.

As shown in Figure 13, a rod connects the middle of the cutter to the stepper motor. With the linear movement of the screw stepper motor, the length Δx changes and causes the rotation of the cutter. The cosine theorem can calculate the length Δx. Before and after each tea plucking process, the rod will return to the zero-point position by running the stepper motor, which means the distance c between the stepper motor and the middle of the cutter can be recorded and read in real time. b is the fixed length of the bracket to hold the cutter and the stepper motor. m is the measured distance from the cutter holder’s rotation point to the cutter’s middle. Consequently, the distance Δx changed by the stepper motor corresponding to α degrees of rotation of the cutter can be calculated as follows:

(4) $$\alpha =\frac{180}{\pi }\cdot \left\{\arccos\left[\frac{b^{2}+r^{2}-{\left(c+\Delta x\right)}^{2}}{2br}\right]+2k\pi \right\}-\frac{180}{\pi }\cdot \left[\arccos\left(\frac{b^{2}+r^{2}-c^{2}}{2br}\right)+2k\pi \right],$$

where k∈Z+. For each image taken by the camera, the above motion logic will be calculated, and the motion will be performed according to the calculation result to meet the real-time requirement. The tea plucking machine walks along the tea canopy and moves the cutter towards the calculated cutting pose simultaneously to realize the cutter profiling of the tea canopy.

## 3. Experimental Results

### 3.1. Comparative Experiment of Different Detection Algorithms

First, under different weather conditions including cloudy, overcast, and sunny, tea canopy images were captured using the Intel RealSense D435i camera. Next, the AG values of the images were calculated and categorized into low-light and high-light images. Finally, the proposed algorithm for tea shoot detection under different lighting conditions was employed to detect the tea shoot, followed by a comparison with other algorithms. To evaluate the performance of the algorithm, this paper selects precision (P), recall (R), F1, and processing speed as the evaluation metrics for the detection performance.

Precision represents the proportion of true positive samples among the samples that have positive prediction results. The formula can be expressed as follows:

(5) $$P=\frac{TP}{TP+FP}$$

Recall represents the proportion of actual positive samples to all positive samples in the entire sample set that are correctly identified as positive by the model. The formula can be expressed as follows:

(6) $$R=\frac{TP}{TP+FN}$$

where TP, FP, and FN are the number of true positive cases, false positive cases, and false negative cases.

The F1 score represents the weighted average of the precision and recall. The formula can be expressed as follows:

(7) $$F1=2\cdot \frac{P\cdot R}{P+R}$$

Precision reflects the algorithm’s ability to distinguish negative samples. The higher the precision, the better the algorithm’s ability to distinguish negative samples. Recall reflects the algorithm’s ability to recognize positive samples. The higher the recall, the better the algorithm’s ability to recognize positive samples. The F1 score combines both the precision and recall. The higher the F1 score, the more robust the algorithm.

**Figure 14:**
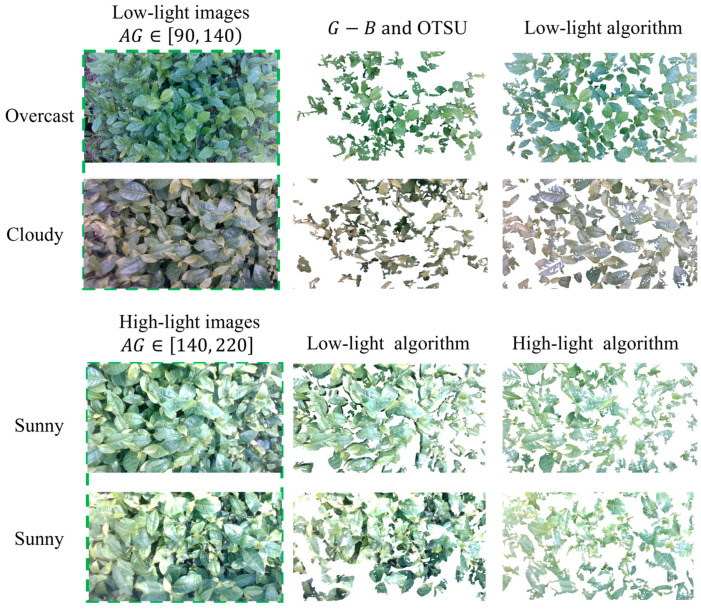
Tea shoot detection results.

As illustrated in Figure 14 and Table 1, the algorithm based on G−B and Otsu could only detect tea shoots with prominently distinct color features. The detection accuracy was lower than our method, with a low quantity of detected tea shoots, most of which were incomplete. When the tea shoot detection algorithm for low-light images was applied to high-light images with specular reflection regions, the resulting detection may include some old leaves and areas affected by specular reflection, as illustrated in Figure 14. In contrast, the tea shoot detection algorithm of the high-light image proposed in this paper could circumvent specular reflection regions and perform Otsu only on non-specular reflection regions. Consequently, there were no specular reflection regions in the detection results, and detection results were improved.

**Figure 15:**
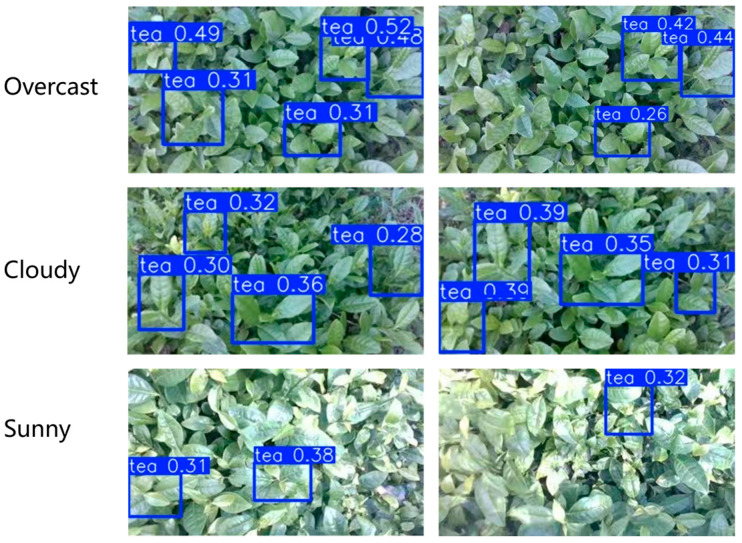
Results of the YOLOv8n model test.

Using the same dataset to train YOLOv8n for detection, as shown in Figure 15 and Table 1, the detection precision of tea shoots was not high, with many missed detections. This is because the shape characteristics of bulk tea shoots and old leaves are not significantly different, and there are only gradual green color differences between them. Additionally, labeling is relatively difficult, which greatly affects subsequent depth information extraction. In contrast, the high-light image tea shoot detection algorithm proposed in this paper can effectively extract young leaf regions, and the detection precision is significantly improved.

### 3.2. Specific Regions Selection Criteria

The selection of two fixed regions in the tea canopy image determines $l_{near}$ and $l_{far}$, which in turn affects the accuracy and robustness of the cutting pose calculation. In order to further determine the selection criteria for specific regions, we conducted experimental analysis with sample 3 of Figure 17. Due to the different impacts of changing the row height and column width on $l_{near}$ and $l_{far}$, we recorded the values of $l_{near}$ and $l_{far}$ when the row height and column width of the regions were varied. As shown in Table 2 and Table 3, we first fixed the row height and divided the entire image into 14 columns to ensure that each column’s pixel width included at least one tea leaf. The regions between different columns were taken for depth value calculation. It can be observed that $l_{near}$ decreased with decreasing column width due to the presence of tea shoots in the center of the image that were significantly higher than the height of the tea canopy. To ensure that the cutting pose adjustment was not too sensitive to individual tea shoots, particularly on irregular tea canopy surfaces, it was advisable to avoid using excessively small column widths. Similarly, choosing too large a column width would reduce the $l_{far}$ due to the effects of tea canopy curvature and camera edge distortion.

On the other hand, as shown in Table 4, when the column width was fixed and the row height increased, it could be observed that the impact on $l_{near}$ was minimal. This was because the depth difference in the tea shoots within the fixed column width range was not significant. Considering the surface undulations on the tea canopy, a larger row height should be chosen for the $l_{near}$ region to ensure robustness and flexibility in adjusting the cutting pose. The $l_{far}$ region had some differences compared to the $l_{near}$ region. As the row height increased, $l_{far}$ decreased. This was because the overall height of the tea tree became tilted, making it difficult to ensure the same horizontal plane in the $l_{far}$ region. Therefore, the row height for $l_{far}$ should not be excessively large.

Considering the variable and complex nature of open-field environments, to ensure good sensitivity and a certain level of robustness in the cutting process, we selected specific ranges for the column width and row height of the central and left regions in this study. For the central region, the column width was chosen to be between 4/14 and 10/14, and the row height was between 3/8 and 5/8. For the left region, the column width was between 1/14 and 2/14, and the row height was between 3/8 and 5/8. These selections aimed to strike a balance between sensitivity and robustness while adjusting the cutting pose in challenging field conditions.

### 3.3. Results of Camera Mounting Angle Experiment

**Figure 16:**
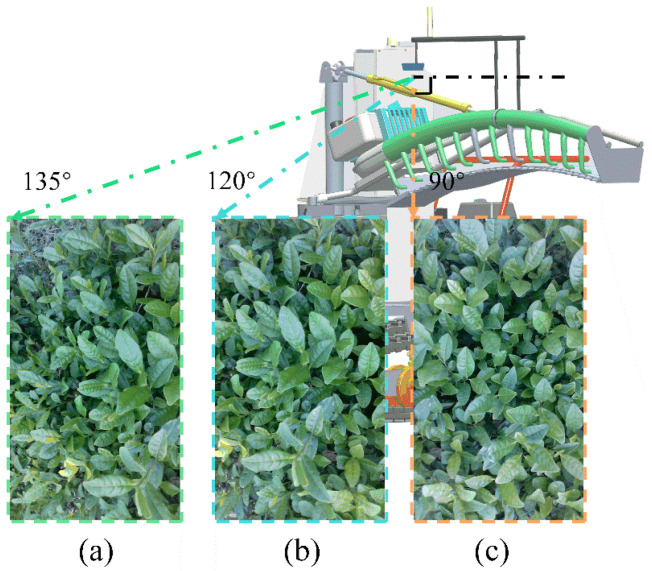
Three installation angles of the RGB-D camera: (**a**) 135°, (**b**) 120°, and (**c**) 90°. In addition, the cutter should be avoided within the camera’s shooting range.

As previously mentioned, the depth camera was mounted on the cutter and moved with it. In order to facilitate the image processing process, only the surface portion of the canopy should appear within the acquired data. Considering the effects of light, reflection, and tea growth characteristics, different camera mounting angles would result in different depth information acquisition effects. When the camera shot the tea canopy at an orientation greater than 90°, more bulk tea shoots were stacked on top of each other, which affected the value of $l_{near}$ and $l_{far}$. As shown in Figure 16, three installation angles of 90°, 120°, 135° degrees were selected for the experiment. The measured vertical distances between the camera and the tea canopy were roughly $l_{near}=45$ cm and $l_{far}=50.3$ cm, respectively. The expected cutting pose could be obtained as $\left(0,\;0.016\right)$ with the known distance from the camera to the cutter. The corresponding results are shown in Table 5. It could be seen that when the camera shot the tea tree top vertically, it could obtain more accurate depth information of the tea canopy and less error in the cutter pose. In addition, the rotation of our tea plucking machine cutter by 0.1 degree roughly required the expansion and contraction of the stepper motor by 2 cm. During each harvesting process, height adjustment remains the primary task. However, the inclination of the machine due to the hilly terrain and the unevenness of the uneven growth of tea canopies lead to the necessity of angle adjustment.

### 3.4. Performance of the Cutter Profiling Method

**Figure 17:**
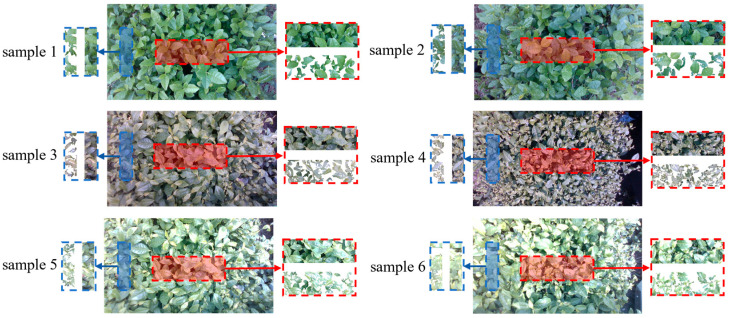
Detection results of tea shoots in $l_{near}$ and $l_{far}$ regions.

By performing our method to retain only the depth information of tea shoots after removing the background and old leaves, we could improve the performance of pose calculation by enhancing the shape matching ability. As described in Section 3.2, to avoid errors caused by calculating the depth value of individual pixels, we chose to calculate the cutting pose based on the average depth of all pixels within a selected region. Figure 17 illustrates the process of tea shoot detection for six samples under three different weather conditions. Table 6 presents the original images and the pose calculation results using our method. The error represented the absolute difference between the calculated $\left(d,\alpha \right)$ based on the RGB-D image of the tea canopy and the actual measured value of $\left(d,\alpha \right)$. It could be observed that our algorithm provides a more accurate calculation of the cutting pose compared to using the original images under different lighting conditions and shooting distances. The presence of depth values influenced by old leaves led to larger errors in the pose estimation results obtained from the raw images.

### 3.5. Field Test Validation

**Figure 18:**
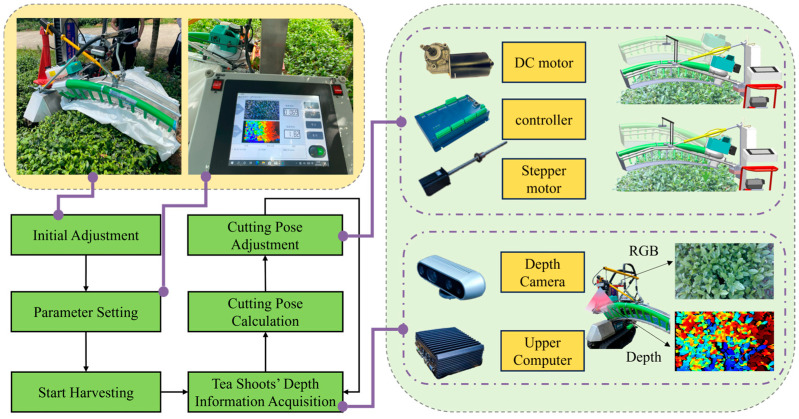
Field test of bulk tea harvesting.

A bulk tea shoots plucking prototype equipped with an adaptive cutter profiling system was established, see Figure 3c. To evaluate the performance of the profiling tea plucking machine, the field test was conducted at the Mechanized Plucking Test Base of Wenjun Tea Plantation, Qionglai, Sichuan Province, China (Figure 3a). The harvesting objects were bulk green tea in five tea samples with a length of 20 m of basically consistent growth. The width and height of the canopy were about 110 cm and 80 cm, respectively. In the test, the plucking machine held the other side of the arc-shaped reciprocating cutter to accomplish the adaptive profiling of the tea canopy. The tea plucking machine was first moved to the tea canopy, and the expected length of tea shoots was set as 10±2 cm through the human-machine interface (Figure 18). We then confirmed that the cutter could automatically descend to the bud layer 10 cm below the tea tree top before running the cutter. Finally, an operator could accomplish the rest tea harvesting process by merely holding one side of the hand-assisted self-propelled plucking machine.

According to the standard of operating quality for tea plucking machines, we utilized the diagonal quartering method to extract 300 g samples from each harvesting and then recorded the bud and leaf integrity rate [12]. The loss rate calculation required the collection of cut but uncollected buds and leaves after harvesting. The uncut buds and leaves in the tea canopies after harvesting were collected to calculate the leakage rate. Furthermore, the results profiling plucking could be evaluated based on the length of the harvested tea shoots, so we also recorded the qualified rate for the length of buds and leaves that met the agronomic requirements of the tea plantation. Bud and leaf integrity rate, leakage rate, loss rate, qualified rate, and hourly productivity per unit width are defined as follows:

(8) $$R_{1}=\frac{W_{1}}{W}\times 100,$$

(9) $$R_{2}=\frac{W_{3}}{W_{2}+W_{3}}\times 100,$$

(10) $$R_{3}=\frac{W_{4}}{W_{2}+W_{3}+W_{4}}\times 100,$$

(11) $$R_{4}=\frac{W_{5}}{W}\times 100,$$

(12) $$E=\frac{\sum A}{\sum TB},$$

where $R_{1}$ represents the bud and leaf integrity rate. W and $W_{1}$ are the total mass of the samples and the mass of complete buds and leaves, respectively. $R_{2}$ and $R_{3}$ indicate the loss rate and leakage rate, respectively. $W_{2}$, $W_{3}$, and $W_{4}$ are the total mass of the samples, the total mass of the dropped buds and leaves, and the total mass of the uncut buds and leaves in several test areas, respectively. $R_{4}$ represents the qualified rate, and $W_{5}$ is the total mass of buds and leaves that meet the length requirement. E is hourly productivity per unit width of the tea plucking machine. A, T, B are the operating area, operating time, and cutting width of the tea tree trimmer, respectively. The average operating time of the tea plucking machine in this field test was 45.4 s.

**Figure 19:**
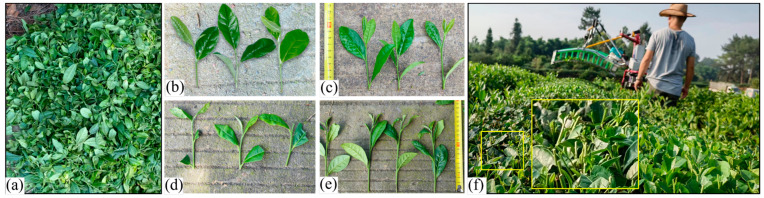
Harvesting results: (**a**) plucked tea leaves, (**b**) buds and leaves that meet the standards, (**c**) buds and leaves of the required length, (**d**) broken leaves, (**e**) buds and leaves that exceed length requirements and (**f**) neat surface on the tea canopy after harvesting.

After the harvesting, the tea canopy’s surface was neat. Meanwhile, the cut of the tea branches was smooth (Figure 19f). Due to reciprocating cutter’s structural constraints, some tea buds and leaves will inevitably be broken. As shown in Table 7, the average rates of intact buds and leaves exceeded 70%, the average rate of missed collection was less than 1.5%, and the average missed harvest rate was less than 2%. In addition, the leakage rate (standard deviation: 0.11%) and loss rate (standard deviation: 0.079%) had the smallest standard deviations, indicating that these two indicators performed stably across different samples and were less affected by changes in test conditions. These results validated that the proposed profiling method meet the quality standard for tea plucking machines and bulk green tea production requirements. The cutter automatically adjusted the height and angle by the profiling method proposed in this study, resulting in the length of the plucked buds and leaves mostly meeting the agronomic requirements, with the average qualification rate reaching 90.2%. There were performance differences among the five distinct test results. This is because the level of standardized construction in mechanized tea plantations influences the harvesting effectiveness of the adaptive profiling cutting tea harvester to a certain extent. If the tea canopy is not appropriately pruned prior to harvesting, resulting in excessive weeds on its upper surface or uneven growth of young tea leaves, it will be impossible to achieve ideal outcomes when calculating the cutting pose based on the depth information of young leaves. Additionally, if the machine paths are not cleared, the chassis of the tea harvester may tilt instantaneously when passing over obstacles, and the cutting pose cannot be adjusted promptly, leading to suboptimal profiling cutting results. Therefore, improving the construction level of mechanized tea plantations and promoting standardized planting models are crucial. Finally, compared to traditional double-carried harvesting machines that rely on four operators and are limited to harvest up to $3335{\;m}^{2}$ per day, the plucking machine proposed in this study only requires a single operator and can harvest $5336{\;m}^{2}$ per day. This not only reduces labor costs but also significantly improves the efficiency of the tea plucking process.

## 4. Discussion

The main focus of this study is the cutter profiling method of the bulk tea plucking machine. However, it is also necessary to consider other aspects, including how agricultural machines can overcome the vibration effects. The vibration of the tea plucking machine mainly originates from three sources: the engine of the crawler chassis, the cutter’s engine, and the vibration produced during the reciprocating cutting process. In order to reduce mechanical vibrations, we will replace the gasoline engine with an electric drive in the future to reduce vibration levels. In addition, the vibration generated when cutting the tea canopy can not be eliminated entirely but can be improved by implementing measures such as adding rubber shock absorbers to the camera installation position. These measures can alleviate the vibration impact during tea plucking in hilly terrains, thereby ensuring the quality of RGB and Depth images and enhancing the stability and performance of the system.

Different lighting conditions for intelligent agricultural machines with outdoor work requirements cause an overall change in image brightness and a decrease in image contrast. We have proposed a suitable solution to address this issue in Section 2.2. In addition, we can activate the camera’s automatic exposure function, increase the resolution, and define a specific region of interest (ROI) to mitigate the impact of lighting conditions on the algorithm. This approach helps mitigate the adverse effects of excessive brightness and reduced contrast caused by specular reflection, ensuring more reliable and accurate image analysis for the algorithm.

Moreover, the tea plucking machine with a cantilever structure, as opposed to the riding structure, provides better passability and adaptability in hilly terrains. The positioning adjustment mechanism is only required on one side of the cutter, while the riding tea plucking machine requires moving rails on both sides of the cutter. Additionally, the cantilever structure facilitates easy disassembly of the cutting blade and allows operators to observe the tea canopy and the actual cutting effect more conveniently. However, unlike the riding tea plucking machine, the cantilever structure cannot physically shield the sunlight.

Lastly, it is worth mentioning that the qualification rate of buds and leaves is also influenced by tea plantation management. Non-standard curved planting of tea canopies can lead to chaotic growth of tea shoots, and satisfactory harvesting results cannot be obtained using a profiling tea plucking machine. The cutter profiling method of the tea plucking machine described in this study applies to tea plantations with standardized management and demonstrates a certain degree of universality. We are working with local agricultural departments to promote a standardized tea plantation management system to lay the foundation for future standardized management.

## 5. Conclusions

In order to improve the automation of bulk tea harvesting, we proposed an adaptive profiling method that included bulk tea shoot detection, cutter two-dimensional pose calculation and motion control. The experimental results show that the detection algorithm can improve the performance of cutter profiling. Each image of the tea canopy taken by the RGB-D camera will correspond to a certain motion logic, and then the height and angle of the cutter are adjusted to achieve the profiling purpose. Compared with the popular double-carried tea plucking machine, the study significantly improves plucking efficiency. Meanwhile, the field test results verified the profiling method’s performance, and the indicators, such as the rates of intact buds and leaves, met the operating quality standards of tea plucking machines.

The main contributions of this paper are as follows:

(1) An improved super-green feature and the Otsu method were studied for identifying bulk tea shoots under different lighting conditions, so as to meet the requirements of target detection in field environments.

(2) In response to the requirement of automatically adjusting the cutting pose of the cutter, an adaptive profiling cutting strategy based on the depth information of young tea shoots was proposed.

(3) The design and implementation of the adaptive profiling tea harvester system were completed, and field validation was conducted in tea plantations in hilly areas, demonstrating the efficiency and feasibility of the system.

Although there are still some aspects and room to be improved continuously in architecture, this study provides a new idea for the profiling method of bulk tea plucking machines, which greatly alleviates the crisis caused by the shortage of rural labor in tea production. To improve the profiling accuracy in future work, it is necessary to dynamically adjust the areas of two fixed regions according to the cutting area of the cutter.

## Figures and Tables

**Table 1:** Detection effects of different methods.

	Weather	P	R	F1	Processing Speed (s)
G−B and Otsu	Overcast	76.0%	73.5%	75%	0.35
	Cloudy	69.4%	66.2%	68%	0.40
YOLOv8n	Overcast	52.7%	49.3%	46%	0.12
	Cloudy	45.0%	43.2%	44%	0.13
Low-light algorithm	Overcast	97.3%	96.2%	96%	0.16
	Cloudy	96.2%	95.1%	95%	0.14

**Table 2:** The result of $l_{near}$ when fixing the row height and changing the column width.

Region	Measured Value	Column Width				
		2/14–13/14	3/14–11/14	4/14–10/14	5/14–9/14	6/14–8/14
$l_{near}(\mathrm{cm})$	47	50.17	48.65	47.67	45.25	41.23

**Table 3:** The result of $l_{far}$ when fixing the row height and changing the column width.

Region	Measured Value	Column Width				
		1/4–2/4	1/14–3/4	1/14–4/14	2/14–3/14	2/14–4/14
$l_{far}(\mathrm{cm})$	59	58.73	57.53	54.56	55.53	52.06

**Table 4:** The result of $l_{near}$ and $l_{far}$ when fixing the column width and changing the row height.

Region	Measured Value	Row Height			
		3/8–5/8	2/8–6/8	1/8–7/8	0/8–8/8
$l_{near}(\mathrm{cm})$	47	47.36	47.34	47.76	47.34
$l_{far}(\mathrm{cm})$	59	60.80	59.07	58.78	58.77

**Table 5:** Experimental results of three mounting angles of the RGB-D camera.

$\mathrm{Angle}\;\left({}^{\circ}\right)$	$l_{near}\left(\mathrm{cm}\right)$	$l_{far}\left(\mathrm{cm}\right)$	$\left(d,\alpha \right)$	Errors
90	45.9412	51.5316	(−0.9412, −0.012)	(0.9412, 0.028)
120	42.6136	49.3843	(2.3864, 0.038)	(2.3864, 0.022)
135	43.3193	49.4256	(1.6807, 0.037)	(1.6807, 0.021)

**Table 6:** Error evaluation of the calculated cutting poses from the Raw and Ours image of tea canopies at different weather conditions and shooting distances.

	Weather	AG	Dataset		Raw			Ours		
			$l_{near}(\mathrm{cm})$	$l_{far}(\mathrm{cm})$	$l_{near}(\mathrm{cm})$	$l_{far}(\mathrm{cm})$	Errors	$l_{near}(\mathrm{cm})$	$l_{far}(\mathrm{cm})$	Errors
1	Overcast	124	45	49.3	35.1825	34.5679	(9.8175, 7.4043)	43.0867	48.8257	(1.9133, 0.2384)
2		121	45	53.8	37.2954	50.3352	(7.7046, 1.0731)	46.7732	53.3801	(1.7732, 0.211)
3	Cloudless	120	50	57.8	39.2994	44.4692	(10.701, 0.1353)	48.9941	59.5362	(1.0059, 0.8726)
4		115	62	72.4	54.9174	76.2755	(7.0826, 1.9478)	63.3421	70.8058	(1.3421, 0.606)
5	Sunny	166	45	52.1	34.5945	43.3684	(10.406, 3.2827)	46.7803	54.118	(1.7803, 1.0142)
6		171	52	44.6	43.4679	40.8472	(8.5321, 1.8861)	50.3358	45.9273	(1.6642, 0.6671)

**Table 7:** Field test results of the tea plucking machine.

Evaluation Index	Number					Average	Standard Deviation
	1	2	3	4	5		
Bud and leaf integrity rate (%)	81.7	80.3	77.2	81.4	85.6	81.2	2.7
Leakage rate (%)	0.91	0.87	1.12	0.83	0.82	0.91	0.11
Loss rate (%)	0.63	0.55	0.76	0.62	0.74	0.66	0.079
Hourly productivity per unit width ($m^{2}/(m\cdot h)$)	1714.3	1469.4	1894.7	1411.8	1531.9	1531.9	177.16
Qualified rate (%)	90.2	91.3	87.9	89.1	92.6	90.2	1.64
Tea making rate (%)	93.3	94.1	91.6	90.7	95.9	93.1	1.84

## Data Availability

The original contributions presented in this study are included in the article. Further inquiries can be directed to the corresponding author.
